# The role of personality, social economic and prevention strategy effects on health-related quality of life among people living with HIV/AIDS

**DOI:** 10.1186/s40249-021-00890-9

**Published:** 2021-08-03

**Authors:** Xiaowen Wang, Hongbing Luo, Enlong Yao, Renhai Tang, Wenbin Dong, Fuyong Liu, Jun Liang, Huilan Li, Minyang Xiao, Zuyang Zhang, Jin Niu, Lijun Song, Liru Fu, Xuehua Li, Shicong Qian, Qing Guo, Zhizhong Song

**Affiliations:** 1 Yunnan Center for Disease Control and Prevention, No.158, Dongsi Street, Xishan Municipal, Kunming, Yunnan Province China; 2 Honghe Municipal Center for Disease Control and Prevention, Honghe, Yunnan Province China; 3 Dehong Municipal Center for Disease Control and Prevention, Dehong, Yunnan Province China; 4 Yuxi Municipal Center for Disease Control and Prevention, Yuxi, Yunnan Province China; 5 Zhaotong Municipal Center for Disease Control and Prevention, Zhaotong, Yunnan Province China; 6 Kunming Municipal Center for Disease Control and Prevention, Kunming, Yunnan Province China; 7 Puer Municipal Center for Disease Control and Prevention, Puer, Yunnan Province China; 8 Wenshan Municipal Center for Disease Control and Prevention, Wenshan, Yunnan Province China; 9 Lincang Municipal Center for Disease Control and Prevention, Lincang, Yunnan Province China

**Keywords:** Health-related quality of life, HIV/AIDS, Multi-level model, Personality factor, Social economic, Prevention strategy

## Abstract

**Background:**

HIV/AIDS has transformed into a chronic controllable but not yet curable infectious disease as other chronic diseases to some extent. The additional of so called fourth 90% that included the improved health-related quality of life (HRQoL) for people living with HIV (PLWHIV) required solutions beyond antiretroviral therapy and viral load suppression. This study will explore the role of personality, social economic and prevention strategy effection on HRQoL among people living with HIV/AIDS.

**Methods:**

A cross-sectional study was conducted among PLWHIV aged more than 16 years old in the 10 municipalities in Yunnan Province, China from October 2019 to May 2020, enrolling total 1997 participants. Individual-level HRQoL data were measured by 12-item Short Form Health Survey (SF-12) and EuroQol Five Dimensions Questionnaire (EQ-5D-5L). We assembled municipal-level data about social economic from Yunnan Statistical Yearbook in 2020 and strategy practice information from the self-evaluation system. We used the principal component analysis to build the social economic and strategy effect on each area respectively and one-way ANOVA was used to perform univariate analysis to identify the predictors with significant differences. Finally we used multi-level model (MLM) to explore the personality, social economic and strategy effects in health-related quality of life among PLWHIV.

**Results:**

The global score for quality of life measured using EQ-5D-5L had an estimated mean score (standard deviation, *SD*) of 0.901 ± 0.146. The HRQoL score measured using PCS-12 had an estimated mean score (*SD*) of 46.62 ± 8.55. The mean MCS-12 score (*SD*) was estimated to be 47.80 ± 9.71. The area-level predictors explained a proportion of 13.6–17.2% for the between-area variation of the HRQoL scores, regardless of the total HRQoL, physical component and mental component. The impacts of stigma (*P* < 0.01), social support (*P* < 0.001), anxiety (*P* < 0.001), depression (*P* < 0.05) and social economic status (*P* < 0.05) on HRQoL at the individual-level were significantly different. The plots visualized the impact of individual-level factors on a respondent’s HRQoL was modified by the area-level characteristics.

**Conclusions:**

The study identified the possible strategy determinant of individual HRQoL of PLWHIV and also the area effect on HRQoL. Stigma, social support, anxiety, depression and social economic status were the individual-level determinants on HRQoL. These could be a valuable resource for evaluating the overall health of the areas and help improve local decision making.

**Graphic abstract:**

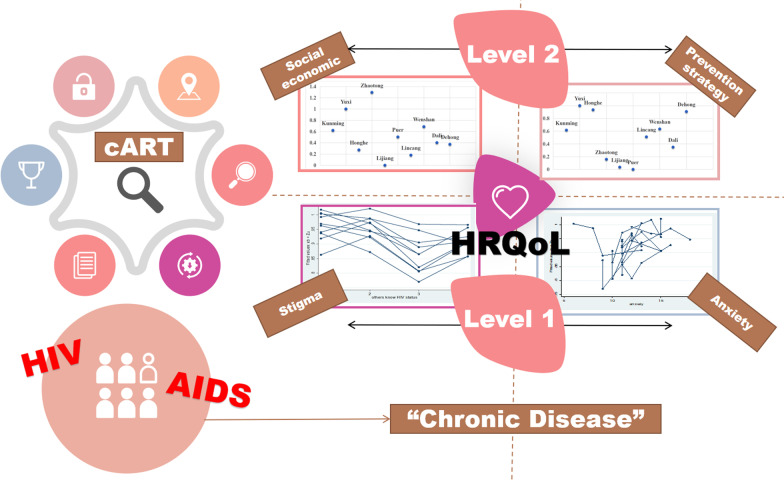

## Background

With advanced access to antiretroviral therapy (ART) and the effective ART delivery of sustained virological suppression, the health outcomes of people living with HIV/AIDS (PLWHIV) have been significantly enhanced [1]. HIV/AIDS has transformed into a chronic, controllable, but-not-yet curable disease similar to other chronic diseases [2]. Health-related quality of life (HRQoL) is a pivotal aspect for both clinical outcomes and research in chronic diseases, which often requires complex and life-long management and therapies [3]. HRQoL points to the self-perceived health of a person with a unitary construct, including several psychological, social, occupational and physical domains, to cope with the complicated progression of chronic diseases [4, 5]. The United Nation UNAIDS 90-90-90 targets (by 2020, 90% of all people living with HIV will know their status, 90% of people diagnosed with HIV infection will receive sustained ART, and 90% of those receiving ART will virally suppressed) [1] are increasingly being achieved in the future. The first-three 90% targets focus on epidemic control, and the addition of the so-called fourth 90% target, which included improved HRQoL for PLWHIV, required solutions beyond ART and viral load suppression [6]. Suitable HRQoL could become equally significant as the first-three targets. We could consider that—exploring the effects of specific factors on HRQoL could help appreciate the quality of health care received and support policy making throughout the entire prevention process.

HRQoL seemed especially impaired among PLWHIV and was associated with many predictors. Previous studies have shown the determinants of HRQoL among PLWHIV. Most of them have emphasized the positive role of ART in improving the HRQoL of PLWHIV [7]. Some social demongraphic factors, such as education level, family economic status and employment status could also contribute to good global HRQoL [8]. Published reviews and meta-analyses have shown insufficient social support, stigma and discrimination, weak adherence to ART, low CD4 + T cell count and advanced HIV stage were linked with poor HRQoL [9, 10]. These factors could be considered as the indictors at the individual level. Some researchers have hypothesized that the individual’s HRQoL is not only defined by their personal-level characteristics, but also socially determined by both physical and social environmental characteristics [11]. This result indicated that we could consider the predictors associated with HRQoL for PLWHIV at both the individual and high levels. Based on the published studies [7–11], we summarized the predictors of HRQoL for PLWHIV into three categories: personality, social economic and strategies. For PLWHIV on ART, the effect of ART was strongly associated with adherence to ART, HIV care clinics and specialized HIV services, which are often defined by the local strategy inclination and professional technology. Otherwise, education level, family economics and employment status could be defined by the area-specific social economic level. Some studies have explored high-level factors to predict dependent variables. One study explored area deprivation, which was an independent effect isolated from the individual effect on adult depressive symptoms in China [12]. One study revealed the family-level factors that influence adolescents’ global physical self- concept. The same result was also found in a study to determine the family clustering of depressive symptoms [13]. For CD4 cell count, a study adopted the same analyses train of thought and demonstrated that the variation existed between patients occupied at 63% and within patients occupied at 37% [14]. One study calculated the proportion of between-county variation in HRQoL that was explained by country-level contextual variables. There were also some studies to adopt the MLM to explore the different level predictors associated with HRQoL in overweight children, cancer patients, adolescents, neuropathic pain patients and cardiovascular patients [5, 15–18]. HIV/AIDS is a disease that requires comprehensive social prevention. Few studies have been conducted to explore the effects on the HRQoL of PLWHIV at different levels, especially at the strategy and area levels, which is critical for policy-making to accomplish the fourth 90% target.

The HRQoL of PLWHIV is an indicator that has multiple dimensions [19], and the effects of predictors associated with HRQoL are always complicated. To guide public health policy, it would be informative to examine the variation in HRQoL among geographic areas and strategy differences. Our study will explore the role of personality, social economic and prevention strategy effects on health-related quality of life among people living with HIV/AIDS and the causal heterogeneity modified by social economic-level and strategy practice-level factors on individual factors to predict HRQoL of PLWHIV to evaluate the overall health in the areas and help improve local decision-making.

## Methods

### Study design and study population

A cross-sectional study was conducted among PLWHIV aged more than 16 years old in the 10 municipalities in Yunnan province, China from October 2019 to May 2020. A convinced sampling method was used. The sample size was calculated by the Yaro Yamane’s approach for finite population using the formula of ${\text{n}} = N/\left( {1 + N(e)^{2} } \right)$ [20]. In our study, n stands for the expected sample size. *N* stands for the finite population that the sample derived from. We set a number of the total PLWHIV estimation of the ten selected areas. e stands for the level of significance and we set 0.05. Finally, the estimated sample size was 354. Based on the reported number of PLWHIVs of each municipality, a convenient sample of 150–200 was included for each selected area. We also excluded respondents with a cognitive impairment who were unwilling to finish the investigation. Our study total included 1997 participants. All investigators from local Center for Disease Control and Prevention (CDC) and social organizations were trained strictly to implement the investigation face to face.

### Data collection

#### Health-related quality of life

Individual-level HRQoL data were measured using the SF-12 and EQ-5D-5L. The 12-item Short Form Health Survey (SF-12), which is the shortened version of 36-item Short Form Health Survey (SF-36) could explain at least 90% of the accuracy of the SF-36 [21]. The SF-12 consists of eight domains to generate two separate summary scores, physical functional scores (PCS) and mental functional scores (MCS) ranging from 0 to 100. Higher scores indicated better HRQoL. Cronbach’s α = 0.89. We also used EQ-5D-5L to measure HRQoL simultaneously. The EQ-5D-5L could define the 3125 possible health states by the different combinations. We adopted the Chinese population-based preference trade-off time (TTO) to transform the measures into utility index (UI, Table 1), thereby producing a single preference-based index ranging from -0.391 to 1.000, where 0 was equal to death and − 0.391 meant worse than death. For example, when we calculated a combination of “21145”, the UI equalled to 1 − 0.066 − 0 − 0 − 0.252 − 0.258 = 0.424 [22]. Cronbach’s α = 0.79.

**Table 1:** Chinese value set for EQ-5D-5L health status

Valuable	EQ-5D-5L
C	–
MO2	0.066
MO3	0.158
MO4	0.287
MO5	0.345
SC2	0.048
SC3	0.116
SC4	0.21
SC5	0.253
UA2	0.045
UA3	0.107
UA4	0.194
UA5	0.233
PD2	0.058
PD3	0.138
PD4	0.252
PD5	0.302
AD2	0.049
AD3	0.118
AD4	0.215
AD5	0.258
N3	–

*EQ-5D-5L EuroQol five dimensions questionnaire, *MO* mobility dimension, *SC* self-care dimension, *UA* usual activities dimension, *PD* pain and discomfort dimension, *UA* pain and discomfort dimension, *N3* constant term

#### Demographic and HIV diagnosis variables

All demographic data, including age, race/ethnicity, education level, marital status, household income per year, whether infection status was known to others, and HIV diagnosis variables including initial infection status, transmission model, duration of ART and the most recent CD4 counts were obtained using self-designed questionnaires.

#### Social support, depression and anxiety

We used the Social Support Rating Scale (SSRS) established by Xiao Shuiyuan in 1986 primarily for the Chinese population [23]. It comprised ten items and three dimensions. A respondent’s social support was measured on three scales: objective social support, subjective social support and support utilization. The final social support was obtained by averaging all item scores from three dimensions. A higher total score demonstrated a higher level of perceived social support. Cronbach’s α = 0.68. Anxiety and depression were measured using Chinese version of the Hospital Anxiety and Depression Scale (HADS) [24], which is a short scale with 14 items designed for anxiety and depression diagnose in nonpsychiatric patients. Anxiety and depression were assessed using seven items respectively. Higher scores demonstrated more serious depression or anxiety symptoms. Cronbach’s α = 0.85.

#### Area-level data collection

We assembled municipal-level social economic data from the Yunnan Statistical Yearbook in 2020 carried out by the Statistical Bureau of Yunnan Province [25]. We used gross domestic product (GDP) per capita, employment rate, birth rate, mortality rate and natural growth rate to calculate the municipal-level social economic effect, which was encouraged to measure the social economic status of the areas. Other municipal-level data about prevention strategy came from the evaluation system for the quality of strategy implemented, which was designed by Yunnan CDC, which included epidemic surveillance, the high-risk behaviour intervention, PLWHIV management, and follow-up and experimental management to construct the prevention strategy. The strategy could be formed different models including of the good quality strategy (stategy 1), the traditional strategy with advantage (strategy 2), the advanced strategy (strategy 3) and the general strategy (strategy 4).

### Data analysis

For the statistical descriptive, we used the mean (standard deviation) and median (interquartile range) to describe the total HRQoL measured using EQ-5D-5L and PCS-12 and MSC-12, respectively.

For the statistical analysis, our study first used five indicators (GDP per capita, employment rate, birth rate, mortality rate and natural growth rate) to demonstrate the social economic effect. All six indicators (the epidemic surveillance score, the comprehensive score of female sex workers intervention, the comprehensive score of men has sex with men intervention, the comprehensive score of PLWHIV management and follow-up and the score of HIV laboratory testing quality) demonstrated the strategy implemented effect of each area. We used principal component analysis to build the social economic and strategy effects of each area. In view of the sensitivity to the dimensions for principal component analysis, all of the calculated indicators were adjusted between 0 and 1 using min–max standardization to eliminate the influence of dimension inconformity [12]. The standardization equation is shown as flowing.

$$S_{ij} = \frac{{x_{ij} - x_{ij(\min )} }}{{x_{ij(\max )} - x_{ij(\min )} }}$$

*S*_*ij*_ demonstrated the transferred *i* indicator of area *j*, *x*_*ij*_ demonstrated the original *i* indicator of area *j*, and *x*_*ij(min)*_ and *x*_*ij(max)*_ demonstrated the max and min *i* indicators in all areas.

We defined the principal components with reference to the variation greater than 80%, and also explanatory variables according to the practice for the social economic effect and strategy effect. In our study, for the social economic and strategy effect, the first and second component scores were calculated as follows:

The first component score = − 0.170 × GDP per capita − 0.229 × employment rate + 0.387 × birth rate + 0.222 × mortality rate + 0.362 × natural growth rate

The second component score = 0.488 × GDP per capita + 0.434 × employment rate + 0.183 × birth rate + 0.322 × mortality rate + 0.112 × natural growth rate

For the strategy practice effect, the first and second component scores were calculated as follows:

The first component score = 0.127 × epidemic surveillance score + 0.343 × the comprehensive score of female sex workers intervention + 0.372 × the comprehensive score of men has sex with men intervention + 0.379 × the comprehensive score of PLWHIV management and follow-up + 0.062 × the score of HIV laboratory testing quality

The second component score = 0.428 × epidemic surveillance score − 0.324 × the comprehensive score of female sex workers intervention + 0.033 × the comprehensive score of men has sex with men intervention + 0.011 × the comprehensive score of PLWHIV management and follow-up + 0.648 × the score of HIV laboratory testing quality

Second, one-way ANOVA was used to perform univariate analysis to identify the predictors with significant differences. Candidates for multivariate analysis included the variables: (1) professionals associated with HRQoL among PLWHIV; (2) social support, anxiety and depression; and (3) variables at the level of *P* less than 0.1 in one-way ANOVA.

Our study used a multilevel model (MLM) to explore the personality, social economic and strategy effects on health-related quality of life among PLWHIV [13, 26]. We set the individual-level as level-1 and the area-level as level-2. Based on the social economic models and strategy practice models by component analyses, we primarily examined the strategy effect as the area-level variables to predict HRQoL, with age, race/ethnicity, marital status, education level, occupation, household income per year, other know HIV status, initial infectious status, transmission model, duration of ART, the most recent CD4 counts, social support score, anxiety score and depression score as individual-level variables to predict the HRQoL. We adapted the random coefficient model to fit. Let *y*_*ij*_ be the score of HRQoL for individual *i* from area *j*. We used one individual-level and one area-level predictor to keep the notation simple and without loss of generality, indicated by *x*_*ij*_ and *z*_*j*_. Respectively. We listed the traditional single-level model as

$$y_{ij} = \beta_{0} + \beta_{1} x_{ij} + \beta_{2} z_{j} + \varepsilon_{ij}$$

Casual heterogeneity was expressed by adding an interaction term, $\beta_{3} x_{ij} z_{j}$, to the model.

For the MLM, the individual-level model includes only individual-level predictors and its regression coefficients were not fixed but varied across areas and fitted into an area-level model.

Individual-level model: $y_{ij} = \beta_{0j} + \beta_{1j} x_{ij} + \varepsilon_{ij}$.

Area-level intercept model: $\beta_{0j} = \beta_{00} + \beta_{01} z_{j} + \mu_{0j}$.

Area-level slope model: $\beta_{1j} = \beta_{10} + \beta_{11} z_{j} + \mu_{1j}$.

The area-level errors $$\left( {\mu_{0j} \mu_{1j} } \right)\sim N\left( {0,\sum { = \left[ \begin{gathered} \tau_{00} \tau_{01} \hfill \\ \tau_{01} \tau_{11} \hfill \\ \end{gathered} \right]} } \right)$$ and were assumed to be independent from the individual-level errors $\varepsilon_{ij} \sim N\left( {0,\sigma^{2} } \right)$. Both the intercept and slope of the individual-level model were determined by the area-level variable. The main effect of area-level variables and the causal heterogeneity were determined by examining the intercept and slope of the individual-level model respectively.

The MLM divided the total variance of HRQoL into between-country (i.e., Σ) and within-country (i.e., σ^2^) variance.

We could also include multiple independent variables in the full MLM such as the multivariate models. In our study, the individual-level independent variables were age, race/ethnicity, education level, household income per year, recent CD4 + T counts, transmission model, duration of ART, social support, anxiety and depression. The area-level predictors in the study were social economic effect and strategy effect.

We used STATA version 14.0 (StataCorp LLC, College Station, TX) to perform all the statistical analysis.

## Results

### The characteristics of subjects

A total of 1997 respondents were enrolled in our study, with a mean age of 45.1 ± 11.8, ranging from 16 to 82. A total of 66.4% of respondents were of Han nationality, and others were from minority ethnic groups, e.g., Yi minority, Zhuang minority, Dai minority and Bai minority. A total of 58.5% of respondents reported themselves as divorced, unmarried and separated. A total of 71.1% of respondents had less than nine years of education. A total of 40.3% of respondents worked as farmers. The average income per capita of respondents’ households was CNY 11 600 in 2020. Regarding their HIV-related characteristics, 70.5% of respondents were in the HIV stage, 28.2% of respondents were in the AIDS stage when they were first diagnosed. A total of 70.2% of samples were those patients with heterosexual transmission, and 18.2% of respondents reported that they had a history of intravenous drug use (IDU). A total of 98.8% of respondents had maintained ART, among those, 58.9% had been treated for more than four years. A total of 71.3% of respondents had high CD4 cell counts (≥ 350 cells/μl) and 50.0% of respondents had CD4 cell counts greater than 500 cells/μl. More details are shown in Table 2.

**Table 2:** The characteristics of our study sample

Characteristic	*n* (%)
Age (years)	
16–18	19 (0.9)
18–30	144 (7.2)
30–45	803 (40.2)
45–60	819 (41.0)
≥ 60	212 (10.6)
Race/ethnicity	
Han nationality	1328 (66.4)
Yi minority	169 (8.4)
Zhuang minority	212 (10.6)
Other minority ethnic group	288 (14.4)
Marital status	
Separated/Divorced/Unmarried	1169 (58.5)
Married/Cohabitating	828 (41.4)
Education level	
< 9 years	1433 (71.7)
≥ 9 years	564 (28.2)
Occupation	
Workers	169 (8.5)
Staff member	124 (6.2)
Farmers	804 (40.3)
Migrant workers	262 (13.1)
Self-employed	358 (17.9)
Unemployed	280 (14.0)
Household income per year (CNY)	
< 5000	627 (31.3)
5000–10 000	600 (30.0)
10 000–50 000	717 (35.9)
≥ 50 000	53 (2.6)
Initial infectious status	
HIV status	1410 (70.6)
AIDS status	565 (28.2)
Unclear	22 (1.2)
Transmission model	
Heterosexual transmission	1403 (70.2)
Homosexual transmission	137 (6.8)
Intravenous drug use	365 (18.2)
Mother-to-infant	23 (1.1)
Unclear	69 (3.4)
Duration of antiretroviral therapy	
≤ 1 years	297 (14.8)
1–2 years	170 (8.5)
2–4 years	328 (16.4)
≥ 4 years	1177 (58.9)
Not yet	25 (1.2)
The most recent CD4 counts	
≥ 500 cells/μl	1000 (50.0)
350–500 cells/μl	423 (21.1)
200–350 cells/μl	356 (17.8)
< 200 cells/μl	189 (9.4)
Unclear	29 (1.4)

### Health-related quality of life

The global quality of life score measured using EQ-5D-5L ranged from -0.391 to 1.000, with an estimated mean score (SD) of 0.901 ± 0.146. The median was 0.951 and the IQR was 0.107. The HRQoL score measured using PCS-12 ranged from 22.63 to 62.31, with an estimated mean score (SD) of 46.62 ± 8.55. The median was 48.22 and the IQR was 12.80. The MCS-12 ranged from 9.70 to 62.66, with an estimated mean score (SD) of 47.80 ± 9.71. The median was 48.19 and the IQR was 13.76. Significant differences in the EQ-5D-5L index score, PCS-12 and MCS-12 were found between age groups (*P* < 0.001), race/ethnicity groups (*P* < 0.05), occupation groups (*P* < 0.001), household income per year groups (*P* < 0.001), others know HIV status groups (*P* < 0.001), transmission model groups (*P* < 0.001) and duration of ART groups (*P* < 0.001). Significant differences in EQ-5D-5L index scores and PCS-12 scores were found between the most recent CD4 counts (*P* < 0.001) and initial infectious status groups (*P* < 0.05). More details are shown in Table 3.

**Table 3:** Health-related quality of life for different variables include in our study

Characteristic	EQ-5D-5L	Physical component score	Mental component score
	Mean (SD)	Mean (SD)	Mean (SD)
Age (years)*			
16–18	0.978 (0.062)	53.54 (4.95)	48.13 (9.76)
18–30	0.948 (0.104)	50.93 (6.95)	49.66 (9.20)
30–45	0.923 (0.125)	48.05 (7.76)	48.17 (9.40)
45–60	0.873 (0.166)	44.76 (8.78)	46.64 (9.86)
≥ 60	0.888 (0.146)	44.90 (9.39)	49.59 (10.13)
Race/ethnicity			
Han nationality	0.895 (0.149)*	45.97 (8.72)*	47.74 (9.64)**
Yi nationality	0.944 (0.105)	48.90 (7.29)	49.85 (10.40)
Zhuang nationality	0.907 (0.117)	48.63 (7.90)	46.74 (9.17)
Other minority ethnic group	0.895 (0.168)	46.83 (8.50)	47.68 (9.89)
Marital status			
Separated/Divorced/Unmarried	0.902 (0.147)	46.24 (8.88)	48.17 (9.77)
Married/Cohabitating	0.899 (0.146)	47.17 (8.04)	47.29 (9.61)
Education level			
< 9 years	0.905 (0.149)	46.71 (8.54)	48.02 (9.63)
≥ 9 years	0.890 (0.139)	46.41 (8.59)	47.25 (9.92)
Occupation*			
Workers	0.906 (0.127)	46.72 (8.30)	46.63 (9.83)
Staff member	0.925 (0.106)	49.49 (7.30)	46.91 (10.31)
Farmers	0.915 (0.125)	47.37 (8.10)	48.87 (9.55)
Migrant workers	0.927 (0.098)	48.50 (8.02)	50.03 (9.46)
Self-employed	0.882 (0.190)	45.60 (8.76)	47.06 (9.61)
Unemployed	0.836 (0.192)	42.18 (9.35)	45.00 (9.22)
Household income per year (CNY)*			
< 5000	0.883 (0.149)	45.41 (8.18)	45.46 (9.59)
5000–10 000	0.879 (0.157)	45.29 (9.16)	46.73 (9.50)
10 000–50 000	0.930 (0.132)	48.53 (8.03)	50.52 (9.39)
≥ 50 000	0.952 (0.067)	50.36 (6.65)	50.78 (8.46)
HIV status known by others*			
No one knowing	0.930 (0.126)	48.71 (7.47)	49.52 (9.21)
Partially	0.914 (0.121)	47.30 (8.26)	47.97 (9.44)
All	0.796 (0.197)	40.01 (8.43)	43.01 (9.81)
Not clear	0.880 (0.168)	44.87 (9.11)	47.61 (10.82)
Initial infectious status			
HIV status	0.908 (0.132)**	47.06 (8.48)*	48.06 (9.62)*
AIDS status	0.890 (0.168)	45.59 (8.58)	47.22 (9.81)
Unclear	0.736 (0.256)	45.59 (10.40)	46.23 (12.34)
Transmission model*			
Heterosexual transmission	0.917 (0.132)	47.899 (7.85)	48.82 (9.57)
Homosexual transmission	0.931 (0.107)	48.34 (7.77)	48.86 (9.89)
Intravenous drug use	0.845 (0.162)	41.80 (8.98)	44.25 (9.00)
Mother-to-infant	0.932 (0.162)	40.95 (9.86)	44.03 (10.26)
Unclear	0.790 (0.238)	40.95 (9.86)	44.03 (10.26)
Duration of antiretroviral therapy*			
≤ 1 year	0.925 (0.124)	47.63 (8.36)	49.24 (9.41)
1–2 years	0.926 (0.094)	47.592 (8.62)	48.58 (9.35)
2–4 years	0.927 (0.110)	48.52 (7.81)	49.91 (9.62)
≥ 4 years	0.883 (0.164)	45.74 (6.98)	46.13 (5.75)
Not yet			
The most recent CD4 counts			
≥ 500 cells/μl	0.902 (0.152)*	47.12 (8.38)*	47.44 (9.83)
350–500 cells/μl	0.909 (0.129)	46.40 (8.22)	48.25 (9.78)
200–350 cells/μl	0.904 (0.130)	46.71 (8.71)	48.02 (9.43)
< 200 cells/μl	0.891 (0.158)	44.80 (9.35)	48.44 (9.51)
Unclear	0.774 (0.216)	43.77 (9.89)	46.92 (9.60)

*P < 0.001, **P < 0.05. *EQ-5D-5L* EuroQol five dimensions questionnaire

### The social economic and strategy effect construction

We adopted principal component analysis to shape the different effects of the ten areas in our study. For the social economic effect, the component analysis showed that the initial eigenvalues of the first and second components were 2.43 and 1.73 respectively, which explained 83.3% of the variance.

**Fig. 1:**
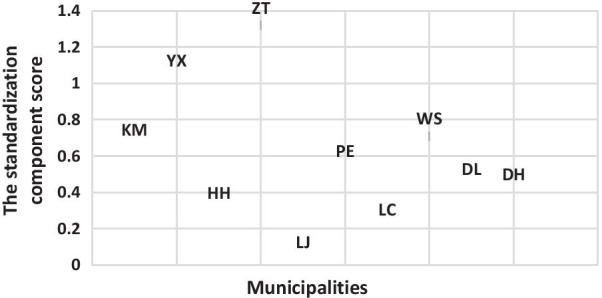
The standardization component score of social economic effect to classify the ten municipalities by four categories

Using standardization, we constructed the social economic effect, as shown in Fig. 1. We considered that the ten areas formed the ten kinds of social economic models.

For the strategy practice effect, the component analysis showed that the initial eigenvalues of the first and second components were 2.38 and 1.41 respectively, which explained 75.8% of variance. When we added the third component, the total variance of 92.1% could be explained, but it is hard to explain the different models of the strategy effect. Thus, we kept the first and second component.

**Fig. 2:**
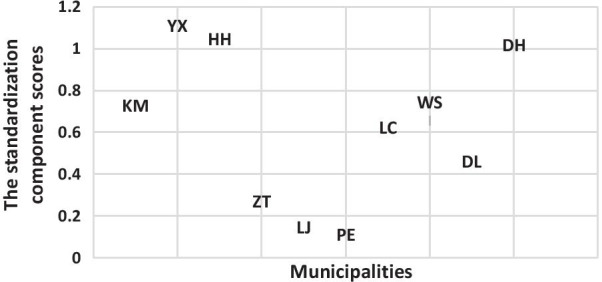
The standardization component score of strategy practice effect to classify the ten municipalities by four categories

Using standardization, we constructed the strategy effect, as shown in Fig. 2. We considered that the ten areas formed four kinds of strategy practice models.

### The effect of area level exploring

Based on the nesting of individual scores into the areas, we began our MLM analyses with the construction of the zero two-level model (area-individual) for HRQoL index score, PCS-12 and MCS-12 to investigate the variance among different areas. We found that the variance for HRQoL index score, PCS-12 and MCS-12 on the area level demonstrated the normal distribution *N*(0, 0.003), *N*(0, 12.7) and *N*(0, 15.1). The variance for all three scores at the area level showed the significant differences (*P* < 0.001). The variance partition coefficients (VPCs) of the HRQoL index score, PCS-12 and MCS-12 were 13.6%, 17.2% and 15.8% respectively. Table 4 shows the partition of variance based on the MLM.

**Table 4:** The partition of variance of Health-related Quality of Life in the MLM analysis

Parameters	_HRQoL index score_	PCS-12	MCS-12
Area-level $\tau_{00}$	0.003	12.689	15.117
Individual-level $\sigma^{2}$	0.019	60.875	80.530
VPCs	13.6%	17.2%	15.8%

*MLM* multi-level model, *HRQoL* health-related quality of life, *PCS* physical functional scores, *MCS* mental functional scores, *VPCs* variance partition coefficients

### Social economic effect, strategy effect and individual effect associated with HRQoL

Regarding the area effect, a significant difference in global HRQoL measured using the EQ-5D-5L index score was found between strategy 1 and strategy 2 (Z = 2.14, *P* < 0.05). A significant difference in the mental component of HRQoL measured using the PCS-12 was found between strategy 1 and strategy 3 (Z = − 3.23, *P* < 0.001).

Regarding individual effects, all the physical component and mental component of the HRQoL measured using PCS-12 and MCS-12 were significantly lower for the group with household income per year less than CNY 5000 compared to the group with household income per year more than CNY 10 000 (*P* < 0.05). A significant difference in global HRQoL measured using EQ-5D-5L was also demonstrated in the variables of all individuals with known HIV status, and unclear if HIV status is known compared to no one knowing the infectious status (*P* < 0.01). A significant difference was found in the physical component of HRQoL measured using PCS-12 between partial known HIV status, all known HIV status and unclear if HIV status is known compared to no one knowing the infectious status (*P* < 0.05). We also found a significant difference between those with known HIV status and those without known HIV status (*P* < 0.01). A significant difference also existed in the variables of anxiety score and depression score (*P* < 0.05). It was demonstrated that the global HRQoL scores increased to 0.010 by decreasing of anxiety scores to 1 and that the global HRQoL scores increased to 0.003 by the decreasing depression scores to 1. These significant differences were also found in the physical component and mental component of the HRQoL measured using PCS-12 and MCS-12 (*P* < 0.05). Otherwise, the social support score contributed positively to predicting the PCS-12 and MCS-12 (*P* < 0.001).

Others, both the physical component and mental component of the HRQoL measured using PCS-12 and MCS-12 were significantly lower for the intravenous drug user group than for the heterosexual transmission group (*P* < 0.05). The physical component of the HRQoL measured using PCS-12 was significantly lower for the group with recent CD4 counts of less than 200 cell/μl compared to the group with recent CD4 counts of more than 500 cell/μl (*P* < 0.05).

Tables 5, 6 and 7 show the details of the predictors at the area-level and individual-level associated with HRQoL, PCS-12 and MCS-12 (Table 8).

**Table 5:** Predictors associated with health-related quality of life of PLWHIV

Characteristic	*β*	SE	Z-value	*P*-value
Strategy practice effect				
Strategy 1	–	–	–	–
Strategy 2*	− 0.042	0.020	− 2.140	0.032
Strategy 3	− 0.012	0.019	− 0.590	0.552
Strategy 4	0.009	0.027	0.320	0.749
Age (years)				
16–18	–	–	–	–
18–30	− 0.045	0.041	− 1.110	0.267
30–45	− 0.051	0.041	− 1.260	0.208
45–60	− 0.078	0.041	− 1.910	0.056
≥ 60*	− 0.097	0.041	− 2.330	0.020
Race/ethnicity				
Han nationality				
Yi minority	0.014	0.011	1.230	0.219
Zhuang minority	0.001	0.011	0.070	0.946
Other minority ethnic group	− 0.002	0.009	− 0.170	0.865
Marital status				
Separated/Divorced/Unmarried	–	–	–	–
Married/Cohabitating	0.001	0.006	0.040	0.967
Education level				
< 9 years	–	–	–	–
≥ 9 years	0.002	0.007	0.230	0.820
Occupation				
Workers	–	–	–	–
Public officers/Staff member	0.010	0.014	0.720	0.474
Farmers	0.015	0.011	1.370	0.171
Migrant workers	0.016	0.013	1.180	0.238
Self-employed	− 0.011	0.012	− 0.940	0.350
Unemployed	− 0.009	0.013	− 0.720	0.474
Household income per year (CNY)				
< 5000	–	–	–	–
5000 to 10 000	− 0.006	0.008	− 0.760	0.449
10 000 to 50 000	0.002	0.008	0.200	0.843
≥ 50 000	0.015	0.019	0.770	0.443
HIV status known by others				
No one knowing	–	–	–	–
Partially	− 0.013	0.007	− 1.81	0.070
All*	− 0.058	0.012	− 4.89	0.001
Not clear*	− 0.036	0.014	− 2.65	0.008
Initial infectious status				
HIV status	–	–	–	–
AIDS status	0.002	0.007	0.290	0.772
Transmission model				
Heterosexual transmission	–	–	–	–
Homosexual transmission	0.006	0.013	0.500	0.617
Intravenous drug use	− 0.010	0.010	− 1.040	0.299
Mother-to-infant	− 0.002	0.037	− 0.060	0.955
Unclear	− 0.055	0.017	− 3.230	0.001
Duration of antiretroviral therapy				
≤ 1 years	–	–	–	–
1–2 years	0.002	0.013	0.180	0.860
2–4 years	0.003	0.011	0.260	0.795
≥ 4 years	− 0.016	0.009	− 1.690	0.091
Not yet	0.029	0.028	1.010	0.310
The most recent CD4 counts				
≥ 500 cells/μl	–	–	–	–
350–500 cells/μl	0.009	0.008	1.120	0.263
200–350 cells/μl	0.003	0.011	0.260	0.766
< 200 cells/μl	− 0.016	0.009	− 1.690	0.091
Unclear	− 0.005	0.040	− 0.120	0.907
Social support	0.001	0.001	1.950	0.052
Anxiety*	− 0.010	0.001	− 7.840	0.001
Depression*	− 0.003	0.001	− 2.580	0.010

*P < 0.05. *PLWHIV* people living with HIV. Strategy 1: the strategy with good practice; Strategy 2: the strategy with traditional advantage practice; Strategy 3: the strategy with advanced practice; Strategy 4: the strategy with the general practice

**Table 6:** Predictors associated with physical component score of health-related quality of life for PLWHIV

Characteristic	*β*	SE	Z-value	*P*-value
Strategy practice effect				
Strategy 1	–	–	–	–
Strategy 2	− 2.753	1.617	− 1.700	0.089
Strategy 3	− 0.357	1.611	− 0.220	0.825
Strategy 4	1.345	2.266	0.590	0.553
Age (years)				
16–18	–	–	–	–
18–30	− 0.660	2.297	− 0.290	0.774
30–45	− 2.286	2.299	− 0.990	0.320
45–60	− 4.030	2.303	− 1.750	0.080
≥ 60*	− 6.539	2.351	− 2.780	0.005
Race/ethnicity				
Han nationality				
Yi minority	0.328	0.626	0.520	0.600
Zhuang minority	0.805	0.634	1.270	0.205
Other minority ethnic group	0.302	0.506	0.600	0.551
Marital status				
Separated/Divorced/Unmarried	–	–	–	–
Married/Cohabitating	0.593	0.362	1.640	0.102
Education level				
< 9 years	–	–	–	–
≥ 9 years	0.686	0.380	1.810	0.071
Occupation				
Workers	–	–	–	–
Public officers/Staff member*	1.854	0.768	2.410	0.016
Farmers	1.002	0.616	1.630	0.104
Migrant workers	1.197	0.752	1.590	0.111
Self-employed	0.149	0.700	0.210	0.831
Unemployed	− 0.144	0.745	− 0.190	0.847
Household income per year (CNY)				
< 5000	–	–	–	–
5000–10 000	0.470	0.440	1.070	0.285
10 000–50 000*	0.997	0.464	2.150	0.032
≥ 50 000	1.625	1.096	1.480	0.138
HIV status known by others				
No one knowing	–	–	–	–
Partially*	− 0.948	0.415	− 2.29	0.022
All*	− 2.376	0.680	− 3.49	0.001
No clear*	− 1.992	0.772	− 2.58	0.010
Initial infectious status				
HIV status	–	–	–	–
AIDS status	0.162	0.398	0.410	0.684
Transmission model				
Heterosexual transmission	–	–	–	–
Homosexual transmission	− 0.389	0.720	− 0.540	0.589
Intravenous drug use*	− 2.083	0.540	− 3.860	0.001
Mother-to-infant	− 3.974	2.117	1.880	0.060
Unclear*	− 2.119	0.975	− 2.170	0.030
Duration of antiretroviral therapy				
≤ 1 years	–	–	–	–
1–2 years	− 0.296	0.720	− 0.410	0.681
2–4 years	0.720	0.606	1.190	0.235
≥ 4 years	− 0.244	0.533	− 0.460	0.647
Not yet	1.472	1.597	0.920	0.357
The most recent CD4 counts				
≥ 500 cells/μl	–	–	–	–
350–500 cells/μl	− 0.330	0.432	− 0.760	0.445
200–350 cells/μl	0.188	0.478	0.390	0.694
< 200 cells/μl *	− 2.046	0.663	− 3.090	0.002
Unclear	− 0.081	2.286	− 0.040	0.972
Social support*	0.086	0.026	3.340	0.001
Anxiety*	− 0.496	0.070	− 7.070	0.001
Depression*	− 0.080	0.069	− 2.980	0.003

*P < 0.05. *PLWHIV* people living with HIV. Strategy1: the strategy with good practice; Strategy 2: the strategy with traditional advantage practice; Strategy 3: the strategy with advanced practice; Strategy 4: the strategy with the general practice

**Table 7:** Predictors associated with mental component score of health-related quality of life for PLWHIV

Characteristic	*β*	SE	Z-value	*P*-value
Strategy practice effect				
Strategy 1	–	–	–	–
Strategy 2	− 0.599	0.928	− 0.620	0.519
Strategy 3*	− 2.967	0.919	− 3.230	0.001
Strategy 4	− 0.523	1.273	− 0.410	0.681
Age (years)				
16–18	–	–	–	–
18–30	− 1.361	2.502	− 0.540	0.586
30–45	− 2.193	2.504	− 0.880	0.381
45–60	− 2.562	2.507	− 1.020	0.307
≥ 60	− 2.105	2.559	− 0.820	0.411
Race/ethnicity				
Han nationality				
Yi minority	0.385	0.679	0.570	0.571
Zhuang minority	− 0.085	0.680	− 0.130	0.900
Other minority ethnic group	0.376	0.546	0.690	0.492
Marital status				
Separated/Divorced/Unmarried	–	–	–	–
Married/Cohabitating	− 0.550	0.394	1.400	0.162
Education level				
< 9 years	–	–	–	–
≥ 9 years	0.551	0.394	-1.400	0.162
Occupation				
Workers	–	–	–	–
Public officers/Staff member	− 0.743	0.836	− 0.890	0.374
Farmers	1.290	0.664	1.940	0.052
Migrant workers	0.949	0.810	1.170	0.241
Self-employed	− 0.468	0.755	− 0.620	0.536
Unemployed	0.232	0.805	0.290	0.774
Household income per year (CNY)				
< 5000	–	–	–	–
5000 to 10 000*	0.986	0.476	2.070	0.038
10 000 to 50 000*	2.232	0.504	4.420	0.001
≥ 50 000	2.236	1.193	1.870	0.061
HIV status known by others				
No one knowing	–	–	–	–
Partially	− 0.874	0.449	− 1.940	0.052
All*	− 2.276	0.720	− 3.160	0.002
No clear	− 1.348	0.840	− 1.600	0.109
Initial infectious status				
HIV status	–	–	–	–
AIDS status	− 0.293	0.432	− 0.680	0.497
Transmission model				
Heterosexual transmission	–	–	–	–
Homosexual transmission	0.234	0.782	0.300	0.764
Intravenous drug use*	− 1.733	0.585	− 2.960	0.003
Mother-to-infant	− 2.224	2.303	− 0.970	0.334
Unclear	− 1.989	1.048	− 1.900	0.058
Duration of antiretroviral therapy				
≤ 1 years	–	–	–	–
1–2 years	− 0.424	0.784	− 0.540	0.589
2–4 years	0.753	0.660	1.140	0.254
≥ 4 years	− 0.652	0.580	− 1.120	0.261
Not yet	− 0.195	1.736	− 0.110	0.910
The most recent CD4 counts				
≥ 500 cells/μl	–	–	–	–
350–500 cells/μl	0.210	0.470	0.450	0.654
200–350 cells/μl	0.106	0.520	0.200	0.839
< 200 cells/μl	0.220	0.720	0.310	0.760
Unclear	2.887	2.490	1.160	0.246
Social support*	0.228	0.028	8.140	0.001
Anxiety*	− 1.243	0.074	− 16.810	0.001
Depression*	− 0.848	0.075	− 11.250	0.001

*P < 0.05. *PLWHIV* people living with HIV. Strategy1: the strategy with good practice; Strategy 2: the strategy with traditional advantage practice; Strategy 3: the strategy with advanced practice; Strategy 4: the strategy with the general practice

**Table 8:** The interaction of selected individual-level predictors and area-level predictors of strategy practice on health-related quality of life for PLWHIV

Characteristic	*β*	SE	Z-value	*P*-value
Health-related quality of life				
HIV infectious status known by others × Strategy practice	− 0.004	0.004	− 1.07	0.286
Transmission model × Strategy practice	− 0.003	0.003	− 0.77	0.440
Anxiety × Strategy practice*	− 2.967	0.001	− 3.32	0.001
Depression × Strategy practice	− 0.002	0.001	− 1.88	0.061
Physical component score				
HIV infectious status known by others × Strategy practice	0.004	0.004	− 1.01	0.311
Transmission model × Strategy practice	− 0.002	0.003	− 0.67	0.502
The most recent CD4 counts × Strategy practice	− 0.003	0.003	− 1.03	0.304
Social support × Strategy practice	0.001	0.004	0.01	0.989
Anxiety × Strategy practice*	− 0.004	0.001	3.28	0.001
Depression × Strategy practice	− 0.002	0.001	1.90	0.057
Mental component score				
HIV infectious status known by others × Strategy practice	− 0.004	0.004	− 1.06	0.291
Transmission model × Strategy practice	− 0.002	0.003	− 0.76	0.449
Social support × Strategy practice	0.001	0.004	0.09	0.925
Anxiety × Strategy practice*	− 0.004	0.001	3.27	0.001
Depression × Strategy practice	− 0.956	0.058	1.87	0.061

*P < 0.001. *PLWHIV* people living with HIV

### The interaction effect on HRQoL at area-level and individual-level

**Fig. 3:**
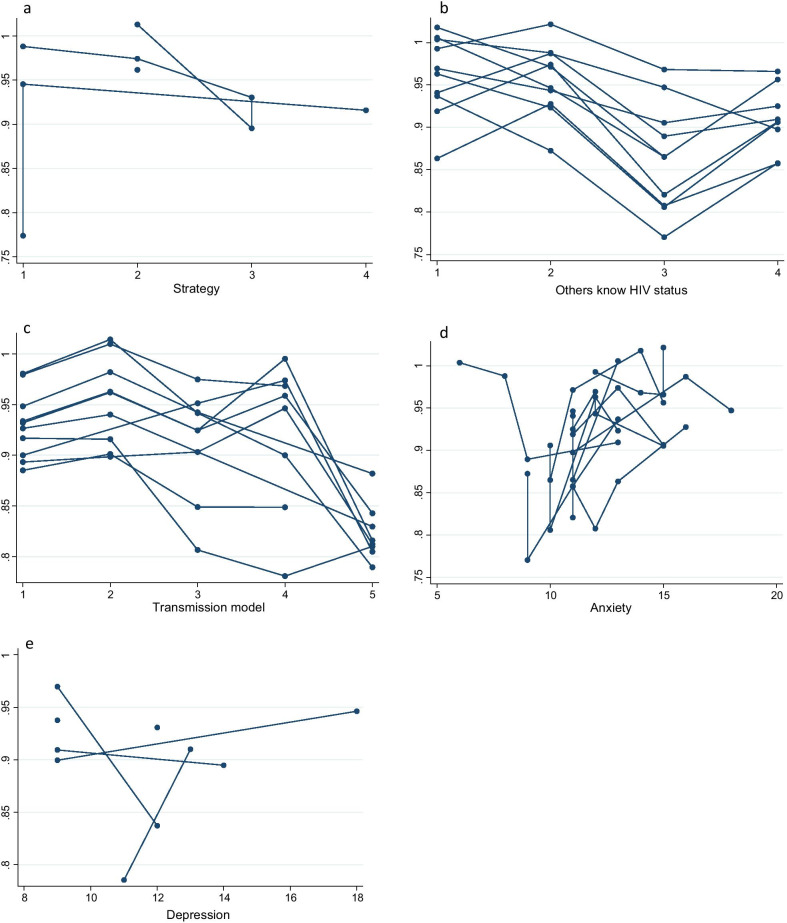
The effect pattern on HRQoL for the five variables (**a** strategy, **b** others know HIV status, **c** transmission model, **d** anxiety, **e** depression). *HRQoL* health-related quality of life

**Fig. 4:**
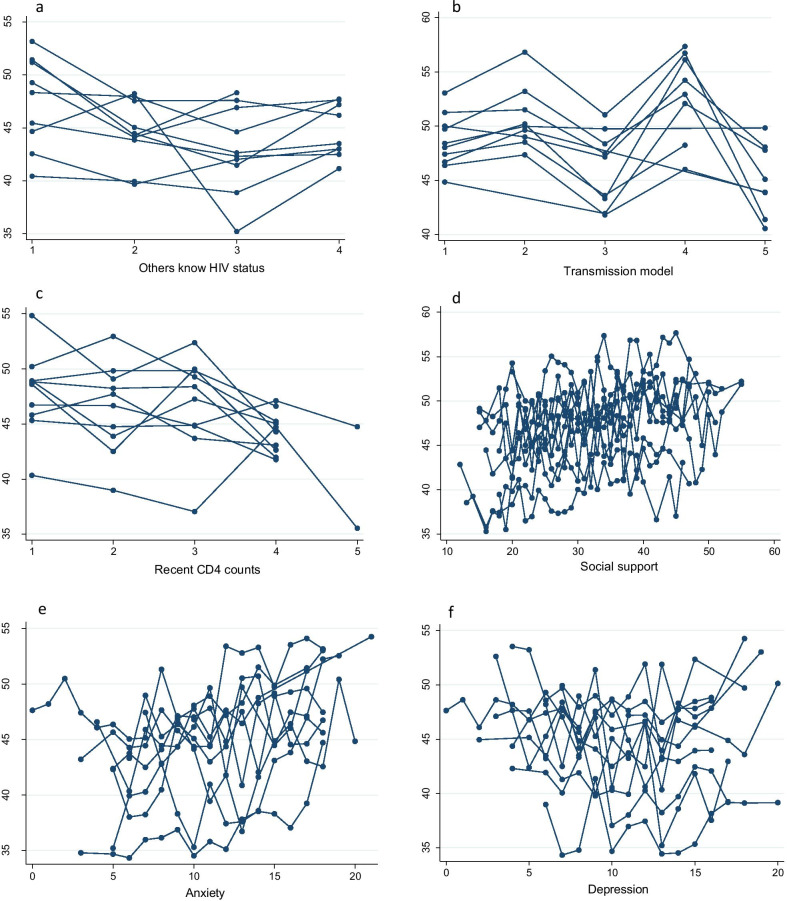
The effect pattern on PCS-12 for the six variables **a** others know HIV status, **b** transmission model, **c** recent CD4 counts, **d** social support, **e** anxiety, **f** depression. *PCS* physical functional scores

**Fig. 5:**
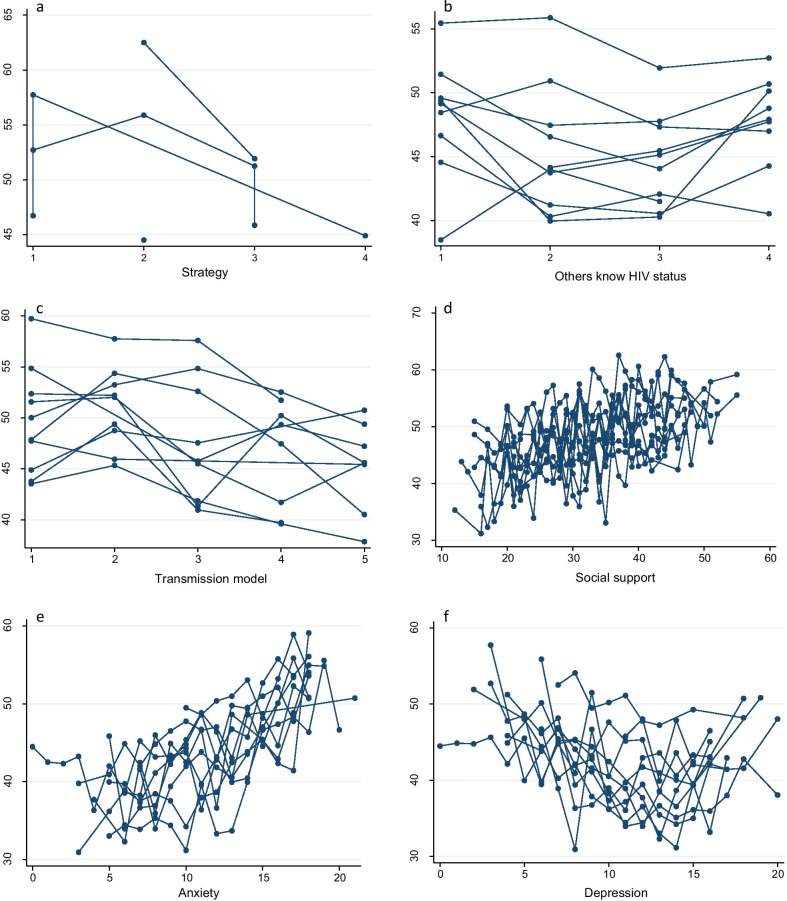
The effect pattern on MCS-12 for the six variables (**a** strategy, **b** others know HIV status, **c** transmission model, **d** social support, **e** anxiety, **f** depression). *MCS* mental functional scores

We calculated the interaction coefficients of the area-level predictor and selected individual-level predictors. The interaction effect is demonstrated between the individual-level variable of anxiety and the area-level variable of strategy practice regardless of global HRQoL or PCS-12 and MCS-12 scores (*P* < 0.001) (Table 7). We also considered the plots to visualize the effects of HRQoL, PCS-12 and MCS-12 scores from selected individual-level predictors modified by area-level effects. Figures 3, 4 and 5 indicate that the variability within and among areas existed in the selected area-level predictor of strategy and in the selected individual-level predictors of other-known-HIV status, transmission model, recent CD4 counts, social support, anxiety and depression. This finding demonstrated that the effects of these variables on HRQoL were different among different areas.

## Discussion

Currently, the HRQoL of PLWHIV has approached a high level, with a mean index score greater than 0.9 measured using the EQ-5D-5L in Yunnan province. However, it is still necessary to explore the predictors associated with HRQoL based on the diversity within and among areas and individuals. Our study applied MLM as an advanced statistical analytical tool to qualify the effect of area-level social economic and prevention strategies and the effect of individual-level variables on the HRQoL of PLWHIV in the era of the fourth 90% target for HIV/AIDS. First, we applied component analyses to classify the social economic model and prevention strategy model for the ten municipalities in Yunnan province. Finally, ten kinds of social economic models were formed based on the GDP per capita, employment rate, birth rate, mortality rate and natural growth rate. By the MLM, we found an area-level effect in ten municipalities on HRQoL, and it was considered that the social economic development status might inform the independent effect on HRQoL, especially on the psychological component of HRQoL. These results were found to be in accordance with one study conducted in China to explore the effect of area deprivation on adult depressive symptoms, which found that area deprivation has an independent contextual effect. Otherwise, the area-level prevention strategy also configured four kinds of models based on the indicators of the epidemic surveillance score, the comprehensive score of female sex workers intervention, the comprehensive score of men who have sex with men intervention, the comprehensive score of PLWHIV management and follow-up and the score of HIV laboratory testing quality. We considered that Kunming, Yuxi and Honghe municipalities informed a strategy that had a good quality to practice the prevention strategy. Dehong municipality kept a strategy practice model of demonstrative plots [27]. Two other kinds of strategy practice models, consisting of Zhaotong, Lijiang, and Puer as well as Wenshan, Lincang, and Dali could be considered general strategy models. In our study, we found that the effect of the second and third strategy models (the Zhaotong, Lijiang, and Puer strategy model as well as the Wenshan, Lincang, and Dali strategy model) on HRQoL and the psychological component of HRQoL decreased compared to the first strategy model (the Kunming, Yuxi and Honghe model). The prevention strategy generally contained early testing and counseling as well as supplying timely and free ART and humanistic care. A good quality of strategy practice could form a good quality prevention system.

Stigma is a traditional predictor in the HIV/AIDS prevention field [28], and can negatively influence happiness, self-esteem, sexual and social relationships and the sense of purpose in PLWHIV [1]. Our study found that whether others know the HIV infectious status has a primary individual-level effect on HRQoL and both the physical component and mental component. An infectious status that some or all of one’s friends orrelatives know could more or less lead to stigma and discrimination so that for a group whose HIV infectious status is open to others demonstrates worse HRQoL. Previous research has shown that HIV-related stigma and discrimination were strongly associated with self-assessed overall HRQoL, especially mental wellbeing [2]. It could also be an obstacle inhabiting the factors to prevent health-seeking behaviour and timely diagnosis. Therefore, one study was conducted to assess the effect of stigma on HRQoL to develop stigma reduction interventions for PLWHIV [1]. One study implemented in children living with HIV/AIDS found that disclosure concerns would be a relevant target for interventions to decrease stigma and improve HRQoL [29].

Social support, anxiety and depression are considered social-psychological characteristics associated with HRQoL [30, 31]. In our study, social support was an individual effect that was positively correlated with the physical and psychological components of HRQoL. Many previous studies have proven the mediation effect to predict the HRQoL of PLWHIV [32, 33]. Good social support has been shown to be a protective resource for improved HRQoL, which was associated with better health outcomes in PLWHIV. Anxiety and depression are the two main mental health metrics that coexist with chronic disease [34]. Our study also showed a negative correlation between depression and anxiety as individual-level effects with HRQoL. Individuals with more serious depression and anxiety symptoms often have worse HRQoL. HRQoL suggests that at least two factors are required [4], one factor assesses physical and functional limitations to be considered as a measure of physical QoL, and the other factor reflects the impact of health on the psychological state to be thought of as a measure of psychological QoL. Generally, psychological influences on HRQoL were considered to be long and powerful when people reported how they felt. Otherwise, the mediation effect among depression, anxiety, social support and HRQoL makes it necessary to advance humanistic care and poverty relief for PLWHIV.

Other the individuals with a higher household income per year contribute to improved physical and mental components of HRQoL, which is consistent with some previous studies [2]. It is not surprising that PLWHIV who had a low income often reported a low HRQoL. There could be a relationship between income and social-economic status, which could facilitate social integration for a better opportunity for health protection and promotion. Our study demonstrated that recent low CD4 counts predicted a poor physical component of HRQoL, the same result found in other studies [3]. This finding emphasizes the importance of timely and effective ART.

The MLM analysis had the greatest advantage of having the ability to fit both the area and individual levels, according to the structure of the data measurement. The main objective of our study was to identify the social-economic and strategy effects at the area-level with impacts on individual HRQoL and quantify the impact. Although predicting HRQoL has some difficulties in obtaining stable perceived measurements, we check the goodness-of-fit using a proportion of between-area variation explained by the country-level predictors. Another advantage of MLMs is that they allow different individual models in different areas. We could examine the initial casual heterogeneity by the relationship between slopes of individual-level effects and area-level variables directly. Therefore, we could determine whether the impact of individual-level factors on a respondent’s HRQoL was modified by some area-level characteristics.

Several limitations were noted in our study. First, adequate area-level variables were hard to acquire; therefore, there were some difficulties in configuring accurate models for social economic and prevention strategies. Second, the effects inhabited in the variables to predict HRQoL were complicated, such as the direct, indirect and mediated effects. Our study did not clarify these relationships. Third, our study did not emphasize more on the variance explanation for the different levels. Fourth, our study did not use a professional measurement scale to define the stigma for PLWHIV. We adopted a variable of whether the HIV status known by others to measure the possible stigma.

## Conclusions

Our study identified the area effect on individual HRQoL of PLWHIV and demonstrated the impact of the stigma, social support, anxiety, depression and social economic status on the HRQoL in the individual-level. The impact of the individual-level factors on a respondent’s HRQoL may be modified by the area-level characteristics to some extents. Our study demonstrated a model combined of area-level social-economic and prevention strategy practice and individual effect on the HRQoL, which could be a valuable resource for evaluating the overall health of the areas and help improve local decision making.

## Data Availability

The data will not be shared because the raw data included the individual’s information, and the information of people living with HIV/AIDS must be kept confidential.

## Abbreviations

ART: Antiretroviral therapy
PLWHIV: People living with HIV/AIDS
HRQoL: Health-related quality of life
SF-12: 12-Item short form health survey
PCS: Physical functional scores
MCS: Mental functional scores
SSRS: Social support rating scale
HADS: Hospital anxiety and depression scale
