# Correction to: The role of personality, social economic and prevention strategy effects on health-related quality of life among people living with HIV/AIDS

**DOI:** 10.1186/s40249-021-00899-0

**Published:** 2021-09-01

**Authors:** Xiaowen Wang, Hongbing Luo, Enlong Yao, Renhai Tang, Wenbin Dong, Fuyong Liu, Jun Liang, Huilan Li, Minyang Xiao, Zuyang Zhang, Jin Niu, Lijun Song, Liru Fu, Xuehua Li, Shicong Qian, Qing Guo, Zhizhong Song

**Affiliations:** 1 Yunnan Center for Disease Control and Prevention, No.158, Dongsi Street, Xishan Municipal, Kunming, Yunnan Province China; 2 Honghe Municipal Center for Disease Control and Prevention, Honghe, Yunnan Province China; 3 Dehong Municipal Center for Disease Control and Prevention, Dehong, Yunnan Province China; 4 Yuxi Municipal Center for Disease Control and Prevention, Yuxi, Yunnan Province China; 5 Zhaotong Municipal Center for Disease Control and Prevention, Zhaotong, Yunnan Province China; 6 Kunming Municipal Center for Disease Control and Prevention, Kunming, Yunnan Province China; 7 Puer Municipal Center for Disease Control and Prevention, Puer, Yunnan Province China; 8 Wenshan Municipal Center for Disease Control and Prevention, Wenshan, Yunnan Province China; 9 Lincang Municipal Center for Disease Control and Prevention, Lincang, Yunnan Province China

## Correction to: Infect Dis Poverty (2021) 10:104 10.1186/s40249-021-00890-9

Following publication of the original article [1], it was found that the authors’ first name and family name are interchanged in the second reference of the article.

The correct reference 2 should be:

- Algaralleh A, Altwalbeh D, Al-Tarawneh F. Health-related quality of life among persons living with HIV/AIDS in Jordan: an exploratory study. HIV/AIDS (Auckland, NZ). 2020;12:897.

The original paper has been updated.
